# Melatonin Prescribing in Adults With Intellectual Disability

**DOI:** 10.1192/bjo.2026.11749

**Published:** 2026-06-30

**Authors:** Ada Ugochukwu, Sthembeni Shoko, Krishna Savadia, Rohit Gumber

**Affiliations:** Leicestershire Partnership NHS Trust, LEICESTER, United Kingdom

## Abstract

**Aims:**

This audit aims to review the prescribing of melatonin in adults with intellectual disability (ID) to understand current patterns of melatonin use within this population and to assess whether prescribing practice follows existing clinical guidelines. In addition, the audit seeks to compare the current findings with those from previous audits conducted in 2018 and 2021 to provide a longitudinal overview of changes in melatonin prescribing over time.

**Methods:**

A retrospective audit was conducted to assess the use of melatonin in adults with intellectual disability and insomnia. From a total population of 322 patients, a sample of 50 patients was selected using systematic random sampling. The inclusion criteria focused on adult patients with intellectual disability prescribed melatonin by the Intellectual Disability Psychiatry Team at Leicestershire Partnership Trust. Patients in transition from the Child and Adolescent Mental Health Services (CAMHS) and those under adult Community Mental Health Teams (CMHT) were excluded. The audit covered the period from January 2022 to December 2024, with data collected from patient records and GP correspondence. Data collection focused on melatonin initiation, dosing, and prior sleep hygiene, with reviews of effectiveness and side effects during the first and most recent 12 months. The later review also assessed withdrawal or dose reduction where appropriate.

**Results:**

Insomnia was the indication for prescription in all 50 patients, with 42% (21/50) having severe, 26% (13/50) moderate, and 32% (16/50) mild Intellectual Disability. The initiation dose was 2mg in 62% (31/50). The current dose is over 10mg (12mg) in 2% (1/50). While all were reviewed post-initiation (within 2 weeks to 1 year), only 40% (20/50) had a review within 2 months. Sleep hygiene advice was undocumented in 80% in the first year and in 88% (44/50) in the past year. There was no review of side effects in 70% in the first 12 months and 51% in the last year. Insomnia had resolved for ≥6 months in 21% (10/50), but 50% (5/10) of these had no trial of dose reduction.

**Conclusion:**

This audit demonstrates some improvement in adherence to prescribing guidelines for melatonin in individuals with intellectual disability, particularly regarding indication, starting dose, and regular reviews of effectiveness. However, it also highlights ongoing gaps in best practice, including timely initial reviews, consistent monitoring of side effects, provision of sleep hygiene advice, and opportunities for deprescribing. These findings underscore the need for more structured and comprehensive approaches to monitoring and documentation.

